# Comparison of STR profiling from low template DNA extracts with and without the consensus profiling method

**DOI:** 10.1186/2041-2223-3-14

**Published:** 2012-07-02

**Authors:** Kelly S Grisedale, Angela van Daal

**Affiliations:** 1 Faculty of Health Sciences and Medicine, Bond University, Gold Coast, QLD, 4229, Australia

**Keywords:** Low template DNA, Stochastic effects, Consensus profiling

## Abstract

**Background:**

The consensus profiling method was introduced to overcome the exaggerated stochastic effects associated with low copy number DNA typing. However, little empirical evidence has been provided which shows that a consensus profile, derived from dividing a sample into separate aliquots and including only alleles seen at least twice, gives the most informative profile, compared to a profile obtained by amplifying the entire low template DNA extract in one reaction. Therefore, this study aimed to investigate the quality of consensus profiles compared to profiles obtained using the whole low template extract for amplification.

**Methods:**

A total of 100 pg and 25 pg DNA samples were amplified with the PowerPlex® ESI 16 Kits using 30 or 34 PCR cycles. A total of 100 pg and 25 pg DNA samples were then divided into three aliquots for a 34-cycle PCR and a consensus profile derived that included alleles that appeared in at least two of the replicates. Profiles from the non-split samples were compared to the consensus profiles focusing on peak heights, allele drop out, locus drop out and allele drop in.

**Results:**

Performing DNA profiling on non-split extracts produced profiles with a higher percentage of correct loci compared to the consensus profiling technique. Consensus profiling did eliminate any spurious alleles from the final profile. However, there was a notable increase in allele and locus drop out when a LTDNA sample was divided prior to amplification.

**Conclusions:**

The loss of information that occurs when a sample is split for amplification indicates that consensus profiling may not be producing the most informative DNA profile for samples where the template amount is limited.

## Background

Polymerase chain reaction (PCR)-based short tandem repeat (STR) analysis is considered the method of choice for forensic DNA profiling. The prominence of the technology is due to the sensitivity of detection from exponential amplification of target molecules by the PCR and the highly polymorphic nature of STRs [1]. This general method allows for small amounts of DNA, between 200 pg and 2.5 ng, to be analyzed with commercial DNA profiling kits [2-6].

In the late 1990s, the Low Copy Number (LCN) technique was introduced to increase the sensitivity of the PCR so that substantially less DNA could be profiled [7]. With this particular process the number of PCR cycles was increased from 28 to 34 resulting in increased results from single cell DNA analysis [7]. The term LCN is often used interchangeably with Low Template DNA (LTDNA). However, in this paper, LCN will refer specifically to the technique of increasing the number of PCR cycles, whereas LTDNA will refer generically to the analysis of samples with 100 pg or less starting template. Amounts less than 100 pg are considered likely to produce results below the stochastic threshold for standard interpretation [8].

The LCN technique can increase the number of alleles observed in a LTDNA profile. However, interpretation difficulties can arise from the exaggerated stochastic effects associated with low levels of starting template. Such effects are well documented and include heterozygote peak imbalance, allele and locus drop out, increased stutter height and allele drop in [9-13].

To accommodate the inherent stochastic effects of LCN DNA typing, a method of replicate analysis has been adopted (referred to as the ‘Biological Model’). In this model, a sample is divided into separate aliquots, generally two or three, and a consensus profile is derived from the replicates that only includes alleles that appear in two or more of the replicates [9]. This biological method is particularly useful for the elimination of non-repeating spurious alleles that appear in a profile as a result of allele drop in [9-13]. Other methods of replicate analysis, such as dividing the sample into four aliquots and including alleles seen in two of the replicates [11], generating a composite profile that includes all alleles seen in the replicate profiles [14] or pooling the sample aliquots post-PCR for a single capillary electrophoresis (CE) injection, [10] have been suggested as possible alternatives to the original biological model.

Critics of the biological model suggest that splitting an already low level sample into multiple aliquots would increase the stochastic effects seen in LTDNA profiles because fewer template molecules are subject to the PCR process in each reaction. As a result, differences are more likely to be seen in replicates of the “same” sample [15]. Additionally, by creating a consensus profile, valuable information from the replicates can be lost, with one study reporting the loss of approximately one third of the alleles obtained [12]. Therefore, critics of the Biological Model advocate efforts should be made to concentrate LTDNA samples rather than diluting and splitting for replicate analysis [15].

Advocates of the Biological Model maintain that a loss of reproducibility is the normal result of LTDNA profiling and, therefore, it is not the existence of variability, but rather the magnitude and potential consequences of any variability that needs to be assessed and reported [16,17]. It has been stated that replicate analyses are preferable to concentrating a sample as this would not usually increase the overall concentration of DNA above the 100 pg stochastic threshold, with stochastic effects still expected to occur in non-replicated samples [17]. However, little empirical evidence has been provided which shows that splitting a LTDNA extract and creating a consensus profile produces a more accurate STR profile than a concentrated LTDNA sample or vice versa.

This study aims to investigate whether concentrating a sample for LTDNA analysis will result in an increased quality STR profile compared with the current practice of splitting extracts into separate aliquots and constructing a consensus profile from the split sample profiles. Samples with known profiles will be used for all experiments. Profiles from low template samples will be compared to high template reference profiles to assess profile quality. Profile quality will be measured in terms of the presence of allele drop out, locus drop out and allele drop in, as well as an analysis of the peak heights and peak height ratios in profiles obtained using the different analysis methods.

## Methods

### Sample preparation

DNA was extracted from five whole blood samples using the BioRobot EZ1® Workstation with the EZ1® DNA Blood Kits (QIAGEN, Hilden, Germany) according to the manufacturer’s instructions. The resulting extracted DNA was quantitated in triplicate using SensiMix^TM^ High Resolution Melt Kits (Bioline, London, UK) according to the manufacturer’s instructions, on a Rotor-Gene^TM^ 6000 (QIAGEN) real time rotary analyzer. Extracts were diluted to low template levels of 100 pg/μl and 25 pg/μl.

### Short tandem repeat analysis

STR analysis was performed using the PowerPlex® ESI 16 Kits (Promega Corp, Madison, WI, USA). The manufacturer’s protocol recommends 30 PCR cycles. Therefore, the samples subjected to the “Standard Cycle PCR” were amplified for 30 cycles. Samples that were analyzed using the “Increased Cycle PCR” were amplified for 34 cycles. Amplification was performed in 25 μl reaction volumes using a GeneAmp® PCR System 9700 (Life Technologies, Carlsbad, CA, USA).

Two series of reactions were carried out. For the first series, 100 pg or 25 pg of DNA templates were placed into one STR amplification reaction. Samples were amplified with 30 or 34 PCR cycles. For each template amount and cycling condition, the five donor samples were amplified in triplicate, to generate a total of 15 profiles per template amount and cycling protocol. For the second series of reactions, 15 (5 extracts amplified in triplicate) 100 pg and 25 pg samples were divided into 3 aliquots, so that 3 reactions containing approximately 33.3 pg or approximately 8.3 pg of template DNA respectively were performed for each 100 pg or 25 pg sample. Each 33.3 pg and 8.3 pg aliquot was amplified with 34 PCR cycles, giving a total of 45 of each 33.3 pg and 8.3 pg profiles, resulting in 15 consensus profiles for both template amounts. Reference profiles for each of the five donors were obtained using the standard cycling protocol using 500 pg DNA template as recommended by the PowerPlex® ESI 16 manufacturer (Promega Corp.). Electropherograms for all samples were obtained using the 3130 Genetic Analyzer (Life Technologies). For each sample, a loading cocktail of 10 μl Hi-Di^TM^ Formamide (Life Technologies) and 1 μl of CC5 Internal Lane Standard 500 (Promega Corp) was mixed with 1 μl of amplified product and denatured for three minutes at 95°C. After cooling, samples were injected on the 3130 using a 3 kv, 5-second injection as is the recommended PowerPlex® ESI 16 protocol. Data were analyzed using Genemapper ID® software version 3.2.1 (Life Technologies) and PowerPlex® ESI 16 panel and bin files. A detection threshold of 50 RFU was used for analysis of all sample profiles as per Tucker *et al*. [18].

### Profile interpretation

Electropherograms for all LTDNA samples were compared with 500 pg control profiles (the recommended template amount for PowerPlex® ESI 16 Kits), noting peak heights, allele drop out, locus drop out and allele drop in. Peak height ratios were calculated by dividing the height of the smaller peak in a heterozygote pair by the height of the larger peak. A peak height ratio of zero was recorded if one allele in the pair failed to amplify. Peak height ratio averages were calculated in two ways. The first calculation used only the heterozygote loci that showed both alleles. The second calculation used all loci in the first calculation, as well as known heterozygote loci that had a peak height ratio of 0% due to allele drop out. While a single peak, and in effect a 0% peak height ratio, would not normally be evaluated when analyzing an unknown profile, these profiles were obtained from known sources. If the peak height ratios are to be used as a measure of how well both alleles at a locus amplify during the PCR then the 0% peak height ratios are an important indicator of the efficiency of the entire reaction. If both alleles at a heterozygous locus failed to amplify, the locus was not used in calculating the peak height ratio average and median.

Locus specific stutter filters provided by the PowerPlex® ESI 16 manufacturer are as follows: 4% (THO1), 8% (D16S539), 9% (D18S1179), 10% (D2S441), 11% (FGA), 12% (D3S1358 and D10S1248), 14% (D19S433), 15% (D1S1656, vWA and D21S11), 17% (D18S51), 18% (D2S1338), 19% (D12S391) and 25% (D22S1045). Since the profiles were from known single source origins a general stutter threshold of 15% was also applied to samples that were subjected to a standard cycle PCR. Stutter has been shown to increase when measures, such as increasing the number of PCR cycles, are taken to improve the detection of low template samples [9]. To compensate for the increased stutter seen in LCN profiles, a stutter threshold of 20% was applied to samples that underwent the increased cycle PCR, based on the method of Caragine *et al*., who observed 97% of stutter was filtered out using a 20% filter for low template samples amplified with an increased cycle PCR and increased injection conditions [10]. If the peak height of an allele in the −4 stutter position exceeded the relevant threshold it was designated as an allele and categorized as allele drop in. No stutter threshold was set for +4 stutter, and consequently any alleles that were present in the +4 stutter positions were designated as alleles and deemed to be allele drop in.

For each of the replicate samples in the second series of reactions, consensus profiles were constructed based on the method outlined by Caragine *et al*. [10], such that an allele had to be seen in at least two replicates to be included as a true allele in the composite profile.

## Results

The first series of reactions, which amplified 100 pg or 25 pg in a single STR amplification, resulted in 15 profiles at standard cycles and 15 profiles at increased cycles for each starting template amount. Each set of 15 profiles comprised 240 total loci. Of the total loci, 183 (approximately 76%) were heterozygous. The second series of reactions, in which 15 100 pg or 25 pg samples were divided into 3 aliquots for an increased cycle amplification, produced 45 profiles at increased cycles, and as such 15 consensus profiles, for each template amount. Each set of 45 profiles consisted of 720 total loci, with 549 (approximately 76%) of these being heterozygous. Each set of 15 consensus profiles comprised 240 total loci, 183 of which were heterozygous.

### Allele drop out

The amplification of 100 pg of starting template using the standard cycling protocol resulted in profiles with little observable allele drop out. Only six (3%) of the heterozygote loci showed allele drop out, with each drop out event occurring in different profiles (Table 1). When the number of cycles was increased to 34, allele drop out was eliminated. However, when the 100 pg samples were split for amplification, the resulting consensus profiles showed an increase in allele drop out. Of the 15 consensus profiles, 16 examples of allele drop out were seen, representing 9% of the total heterozygote loci. The number of drop out alleles per consensus profile ranged from0 to 4, with an average of 1.73 drop out events per profile.

**Table 1:** Allele drop out (ADO)

	**Number of ADO**	**% Heterozygote Loci with ADO**
**100 pg Starting Template**		
*30 PCR Cycle Amplification*		
Non-split Samples (n^a^ = 183)	6	3%
*34 PCR Cycle Amplification*		
Non-split Samples (n = 183)	0	0%
Split Samples (n = 549)	114	21%
Consensus Profiles (n = 183)	16	9%
**25 pg Starting Template**		
*30 PCR Cycle Amplification*		
Non-split Samples (n = 183)	80	44%
*34 PCR Cycle Amplification*		
Non-split Samples (n = 183)	61	33%
Split Samples (n = 549)	250	46%
Consensus Profiles (n = 183)	92	50%

^a^ n is the total number of heterozygote loci.

Allele drop out significantly increased as the amount of starting template was reduced. Using the standard cycle protocol, amplification of 25 pg of starting template resulted in 80 occurrences of allele drop out over 15 profiles, representing 44% of the total heterozygous loci (Table 1). The number of drop out alleles per profile ranged between 3 and 8, with an average of 5.4 allele drop out events per profile. When the samples were subjected to an increased cycle PCR, the percentage of allele drop out was reduced to 33%, with 61 examples of allele drop out over 15 profiles. The number of drop out alleles in each profile obtained using the increased cycling method ranged between 2 and 7, with an average of 4.07 per profile. However, when 25 pg of starting template was split for amplification the resulting consensus profiles showed an increase in allele drop out, with 92 cases over the 15 consensus profiles, which corresponds to 50% of the total heterozygous loci. The number of drop out alleles per profile ranged between 2 and 10, with an average of 7.67 drop out events in each profile.

### Locus drop out

For the purpose of this study, locus drop out was defined as the single allele from a homozygous locus, or both alleles from a heterozygous locus, missing from the profile. In the latter case, both missing alleles were not each counted individually as allele drop out. Locus drop out was not seen in any of the profiles obtained from 100 pg starting template, regardless of whether the sample was amplified using the standard or increased cycle PCR (Table 2). When the 100 pg samples were divided into three 33 pg aliquots and used to construct a consensus profile, the individual profiles did show some locus drop out, with 12 instances seen across the 720 loci. The consensus profiles derived from the aliquots were complete and correct since locus drop out did not occur at the same locus more than once in any set of three replicate profiles.

**Table 2:** Locus drop out (LDO)

	**Number of LDO**	**% Loci with LDO**
**100 pg Starting Template**		
*30 PCR Cycle Amplification*		
Non-split Samples (n^a^ = 240)	0	0%
*34 PCR Cycle Amplification*		
Non-split Samples (n = 240)	0	0%
Split Samples (n = 720)	12	2%
Consensus Profiles (n = 240)	0	0%
**25 pg Starting Template**		
*30 PCR Cycle Amplification*		
Non-split Samples (n = 240)	51	21%
*34 PCR Cycle Amplification*		
Non-split Samples (n = 240)	13	5%
Split Samples (n = 720)	245	34%
Consensus Profiles (n = 240)	79	33%

^a^ n is the total number of loci.

Locus drop out was much more evident in the 25 pg samples (Table 2). Under standard cycling conditions, 51 examples of locus drop out were recorded over the 240 total loci (21%). Between 0 and 7 loci dropped out per sample, with an average locus drop out of 3.4 per sample. This drop out was reduced when the number of PCR cycles was increased, with only 13 (5%) of the total loci dropping out. Under the increased cycle amplification condition, the number of locus drop out events per sample ranged between none and two, with an average of less than one drop out locus per profile. However, when the samples were split and a consensus profile was derived, locus drop out increased, with 79 instances seen in the 15 consensus profiles, representing 33% of the total loci. The number of drop out loci per sample ranged from 3 to 10, with an average of 5.27 loci dropping out in each consensus profile.

### Allele drop in

Allele drop in was minimal under standard cycling conditions, with only two additional alleles seen across all 100 pg sample profiles, one of which was seen in the −4 stutter position and the other seen in the +4 stutter position (Table 3). Drop in increased when the samples were amplified with the increased cycle PCR, with 32 additional alleles seen in the resulting 15 profiles. The number of drop in alleles per sample ranged between 0 and 4, with an average of 2.13 additional alleles seen in each sample. Allele drop in also occurred in the profiles of the split samples, with a total of 32 additional alleles seen in the 45 split sample profiles. However, the consensus method requirement for an allele to be seen twice effectively counteracted this drop in, so that no additional alleles were seen in the 15 consensus profiles.

**Table 3:** Allele drop in (ADI)

	**Allele Drop In Placement**			**Number of ADI**	**% Loci with ADI**
	**Minus 4**	**Plus 4**	**Random**		
**100 pg Starting Template**					
*30 PCR Cycle Amplification*					
Non-split Samples (n^a^ = 240)	1	1	0	2	0.3%
*34 PCR Cycle Amplification*					
Non-split Samples (n = 240)	2	10	20	32	13%
Split Samples (n = 720)	11	11	10	32	4%
Consensus Profiles (n = 240)	0	0	0	0	0%
**25 pg Starting Template**					
*30 PCR Cycle Amplification*					
Non-split Samples (n = 240)	0	0	0	0	0%
*34 PCR Cycle Amplification*					
Non-split Samples (n = 240)	2	1	3	6	3%
Split Samples (n = 720)	10	1	4	15	2%
Consensus Profiles (n = 240)	0	0	0	0	0%
**Total (n = 2,400)**	**26 (30%)**	**24 (27%)**	**37 (43%)**	**87**	**4%**

^a^ n is the total number of loci.

A similar pattern was observed in the 25 pg sample profiles. No additional alleles were seen in the standard cycle PCR profiles, but 5 drop in alleles were noted in the 15 increased cycle PCR profiles. When the samples were divided into 3 aliquots for amplification, 14 additional alleles were seen in the 45 split sample profiles. However, again, the consensus method eliminated this drop in, so that no additional alleles were seen in the consensus profiles.

Of the 87 additional alleles observed across all profiles, 26 were seen in the ‘-4’ stutter position. Alleles in this position were only counted as drop in if their peak height exceeded the nominated stutter ratio filters (locus specific stutter filters followed by a manual examination using a 15% filter for samples amplified with the standard number of cycles and 20% for samples amplified with an increased cycle PCR). Indeed, 140 additional peaks were actually seen in −4 stutter positions; however, 114 were removed from the final profiles by the stutter filters. Twenty-four additional alleles were observed in ‘+4’ stutter positions. While 23 of the 24 additional alleles in +4 positions had peak heights less than 20% of the true allele, a filter was not set for +4 stutter; therefore, additional alleles in this position were counted as drop in. The remaining 37 additional alleles were placed throughout the profiles.

### Peak heights and peak height ratios

For the 100 pg samples amplified with 30 PCR cycles, the height of homozygous peaks ranged from 185 to 847 Relative Fluorescence Units (RFU), with an average peak height of 520 RFU. The height of heterozygous alleles ranged between 55 and 725 RFU, with an average of 261 RFU (Table 4). The peak height ratio range for heterozygote loci was 16% to 99% with a peak height ratio average of 69%. When taking into account the heterozygote loci that had a peak height ratio of 0% due to allele drop out, the average was reduced to 67% (Table 5).

**Table 4:** Peak heights

	**Homozygous Peaks (RFU)**				**Heterozygous Peaks (RFU)**			
	**n**	**Range**	**Mean**	**Std Dev.**	**n**	**Range**	**Mean**	**Std Dev.**
**100 pg Starting Template**								
*30 PCR Cycle Amplification*								
Non-split Samples	57	185 to 847	520	170	360	55 to 725	261	116
*34 PCR Cycle Amplification*								
Non-split Samples	57	662 to 7,609	4,129	1,767	366	139 to 5,805	1,925	1,233
Split Samples	168	166 to 6,131	1,610	1,090	949	51 to 4,975	763	638
**25 pg Starting Template**								
*30 PCR Cycle Amplification*								
Non-split Samples	49	51 to 337	149	73	200	50 to 360	110	56
*34 PCR Cycle Amplification*								
Non-split Samples	53	212 to 3,475	1,259	805	287	58 to 3,500	708	602
Split Samples	122	59 to 2,144	541	369	458	53 to 2,074	386	260

**Table 5:** Peak height ratios (PHR)

	**ADO (0%) Included In the PHR Calculation**				**ADO (0%) Not Included In the PHR Calculation**			
	**n**^**a**^	**Mean PHR**	**Std. Deviation**	**Median PHR**	**n**^**b**^	**Mean PHR**	**Std. Deviation**	**Median PHR**
**100 pg Starting Template**								
*30 PCR Cycle Amplification*								
Non-split Samples	183	67%	23%	70%	177	69%	20%	71%
*34 PCR Cycle Amplification*								
Non-split Samples	183	65%	22%	69%	183	65%	22%	69%
Split Samples	540	45%	32%	47%	426	57%	24%	55%
**25 pg Starting Template**								
*30 PCR Cycle Amplification*								
Non-split Samples	140	29%	36%	0%	60	68%	18%	65%
*34 PCR Cycle Amplification*								
Non-split Samples	174	36%	32%	38%	113	56%	22%	52%
Split Samples	353	19%	32%	0%	103	64%	24%	60%

^a^ n is the number of heterozygote loci with at least one allele present.

^b^ n is the number of heterozygote loci with both alleles present.

The peak heights increased when the number of PCR cycles was increased to 34. For homozygous alleles, the peak heights ranged between 622 and 7,609 RFU with an average height of 4,129 RFU. The peak height range for alleles at heterozygous loci was 139 to 5,805 RFU with an average height of 1,925 RFU (Table 4). However, increasing the number of PCR cycles resulted in a slightly reduced peak height ratio average of 65%, with a peak height ratio range of 6% to 100% (Table 5). Allele drop out was not seen in any of the 100 pg increased cycle profiles; therefore, only one calculation was performed.

The 100 pg samples that were split for amplification and were subject to 34 PCR cycles displayed peak heights higher than those subjected to the 30-cycle amplification, presumably because the increased number of cycles compensates for the decreased template amount. The heights of homozygous peaks ranged from 166 to 6,131 RFU, with an average height of 1,610 RFU. The heterozygous loci showed a peak height range of 56 to 4,123 RFU, with an average peak height of 763 RFU (Table 4). The peak height ratio range for heterozygous loci showing both alleles was 8% to 99%, with an average of 57%. Inclusion of the 114 heterozygote loci that had a 0% peak height ratio, the average was reduced to 45% (Table 5).

Amplification of 25 pg starting template resulted in a peak height reduction compared with the 100 pg samples. Under standard PCR cycling conditions, the heights of homozygous peaks ranged between 51 and 337 RFU, with an average of 149 RFU. For heterozygous loci, the peak height range was 50 to 360 RFU, with an average height of 190 RFU (Table 4). The peak height ratios range for the heterozygous loci was 35% to 99% with an average 68%. When all heterozygous loci that showed at least one allele were included in the calculation, the average peak height ratio was reduced to 29% (Table 5).

As with the 100 pg samples, by increasing the number of PCR cycles the average peak height for the 25 pg samples also increased. For homozygous alleles, the average peak height was 1,259 RFU, with a range of 212 to 3,475 RFU. The height of heterozygous alleles ranged between 58 and 3,500 RFU, with an average height of 708 RFU (Table 4). Considering only the heterozygous loci that showed both alleles, the peak height ratio range was 10% to 99%, resulting in a peak height ratio average of 56%, a reduction compared to the average of the standard cycle samples. However, when the heterozygous loci that had a peak height ratio of 0% were included in the average calculation, the peak height ratio average was higher compared to the standard cycle profiles at 36%, due to the reduction in allele drop out (Table 5).

For the 25 pg samples split for amplification, the heights of the alleles were increased compared with the standard cycle amplification samples due to the increased number of PCR cycles utilized. For homozygous alleles, the peak heights ranged from 59 to 2,144 RFU; however, the average height was only 541 RFU. The range for heterozygous allele heights was similar at 53 to 2,077 RFU, with an average height of 386 RFU (Table 4). The peak height ratio range of heterozygous loci showing both alleles was 16% to 100%, with an average of 64%. However, when the 250 heterozygous loci that had a peak height ratio of 0% were included in the calculation, the average was reduced to 19% (Table 5).

## Discussion

This study supports previous studies that showed increasing the number of PCR cycles will increase the sensitivity of detection for STR profiling of LTDNA samples. However, while 100 pg has been noted as the upper limit for what may be considered a low template sample [7,8], this research shows that when using the current generation multiplex kits, there may be less benefit to increasing the number of PCR cycles when this amount of DNA template is available for amplification. When 100 pg of template was amplified using the PowerPlex® ESI 16 kit with an increased cycle PCR additional alleles were seen in the profiles. This is not surprising due to the increased sensitivity of the PowerPlex® ESI 16 kit, which has been shown to produce full profiles down to 62.5 pg using its standard cycling protocol [18]. Furthermore, the standard protocol for this kit already utilizes 30 PCR cycles, as opposed to other commercially produced multiplex kits that use 28 or 29 cycles as the standard cycle number.

For the 100 pg samples amplified using the standard cycling protocol, there were only 6 instances of allele drop out in the resulting 15 profiles, no locus drop out and only 2 spurious alleles seen overall. Of the two additional alleles, one was in the −4 position and one was in the +4 position to true alleles. When measured against their respective true alleles, the allele in the −4 position had a peak height ratio of 26%, while the allele in the +4 position had a peak height ratio of 36%. The position of the alleles could indicate that they are increased stutter rather than true drop in alleles. However, given the height of the additional peaks, particularly the +4 allele, it may be that they are true drop in. Furthermore, the additional alleles occurred at heterozygous loci, and in both cases the two correct alleles were also present. The presence of the ‘drop in’ alleles would normally indicate a potential mixture sample, with the additional alleles not part of the major profile. However, the other 14 loci showed no additional minor alleles, which would indicate the likely drop in or artifact nature of these allele peaks. In comparison, the 100 pg samples amplified with 34 cycles did not display any drop out. However, 32 additional peaks were seen across the 15 profiles, the majority of which were not in the stutter positions. Of the additional alleles, 10 were seen at homozygous loci, which could mean that the loci could be falsely interpreted as heterozygote loci. However, given that the peak height ratios of nine of the additional alleles was less than 3% of the true allele, this is unlikely. One drop in allele had a peak height ratio of 10% compared to the true allele, but this additional allele was seen in the +4 stutter position, and would also be interpreted with caution. Overall, these results suggest that performing one standard cycle PCR is preferable to performing one increased cycle reaction when 100 pg of template are available for amplification because of the large reduction in allele drop in.

When the template amount is reduced, this research demonstrates that there is significant benefit to increasing the number of PCR cycles for STR typing in terms of the increased amount of information seen in the resulting profiles. With a starting template amount of 25 pg, increasing the number of PCR cycles resulted in a 22% increase in the number of correct loci seen overall compared to the standard cycle profiles. Both allele and locus drop out were markedly reduced and the peak heights and peak height ratios both improved. However, as with the 100 pg samples, increasing the number of PCR cycles for the amplification of 25 pg did result in more drop in alleles seen in the profiles. Compared to the standard cycle profiles, which did not show any additional alleles, the increased cycle profiles displayed six loci with additional alleles. Of these additional alleles, four were seen at heterozygous loci, with three of these loci also containing both true alleles. The fourth locus did display allele drop out in conjunction with the allele drop in, so it is possible this locus would be interpreted incorrectly. The peak height ratio of the drop in allele compared to the remaining allele was only 9%, indicating that this would be interpreted with caution. However, if the drop in allele was considered not part of the major profile, the locus could then be falsely interpreted as homozygous. Of the two additional alleles that occurred at homozygous loci, one occurred in the −4 stutter position but was not removed by the stutter filter as it had a peak height ratio of 29%. The other was in the +4 stutter position, with a peak height ratio of 7%. If an increased cycle procedure were implemented for LTDNA amounts as low as 25 pg, the profile interpretation would need to accommodate the chance of an allele resulting from allele drop in.

For both starting template amounts, the data show that splitting the sample into three aliquots and constructing a consensus profile did not result in the most informative profile compared with a profile where the DNA extract was amplified in one reaction. While the consensus profile approach did eliminate allele drop in, all other measures of profile quality were improved when the sample was not split. The original purpose of the Biological Model approach was to eliminate spurious alleles from the final consensus profile and give confidence that the final profile contains only the alleles of the actual contributor. The former was demonstrated by our results, with no additional alleles in the consensus profiles. This is important, as additional alleles in the profile can result in an incorrect interpretation, where either a homozygous locus is interpreted as a heterozygous locus or, if the drop in occurs in conjunction with a drop out, the wrong genotype may be assigned for that locus. This could have serious ramifications for casework, as errors in the profile could then lead to false inclusion or exclusion of suspects, or false matches if the profile is subjected to a database search.

The consensus profile results showed that a large amount of information was lost when the starting template was divided for amplification. This was especially evident in the 25 pg samples. Compared to the profiles obtained with the full 25 pg using a 34-cycle reaction, the consensus profiles showed a notable increase in allele and locus drop out. When the entire sample was amplified with 34 PCR cycles, 67% of the loci were complete and correct, 25% were heterozygous loci that showed only one allele, 5% showed complete locus drop out and 3% showed allele drop in. When the 25 pg was split for amplification, the resulting consensus profile showed only 29% of the loci as complete and correct, 38% were heterozygous loci that showed only one allele and 33% displayed complete locus drop out. This loss of information that occurs when the sample is divided for amplification is not surprising, since the starting template amount in each of the split samples is barely more than the DNA that is available from a single cell, and that is only if the starting 25 pg is divided equally into thirds. In reality, any of the three aliquots could contain less than a single copy of the genome. Furthermore, if the majority of the 25 pg template happens to end up in one aliquot, then the consensus profiling method may effectively eliminate much information that would be gained from that aliquot’s profile because it does not appear in one of the other aliquot profiles. While repeatability is an important measure of reliability, the fact that so much information is lost in the attempt to repeat the results would suggest that the consensus profiling method may not be giving the most informative profile for samples with such a low level of starting template. It could then be argued that the gain of having confidence that no allele drop in has occurred is not sufficient compensation for the loss of profile information. This is particularly so given the increased occurrence of stutter in the split profiles, since a stutter and drop in are in fact both incorrect alleles. The results from the 25 pg split samples show 10 additional alleles in the −4 stutter position, 1 in the +4 stutter position and 4 in other positions. As a consequence, many of the incorrect alleles being eliminated from the consensus profile are likely increased stutter, which is not actually seen in such high amounts in the non-split profiles (six additional alleles overall, two of which are in the −4 stutter position, one in the +4 stutter position, and three in random positions). Based on the allele drop in results, the use of a higher stutter filter for the −4 position and implementation of a +4 stutter filter would significantly reduce the incidence of apparent allele drop in. Therefore, a profile interpretation method that accommodates the increased stutter may be warranted.

It should be noted that, since allele and locus drop out and allele drop in still occurred when a low template DNA sample was amplified in a single reaction, a robust statistical analysis model that takes the stochastic effects into consideration must be applied to the data. A statistical analysis taking these stochastic effects into consideration should, of course, also be applied to the consensus profiles. It is noteworthy that this study was confined to single source samples. Interpretation of mixture profiles generated from LTDNA samples deriving from more than one individual would be more complex. Sample degradation or the presence of PCR inhibitors – issues commonly seen with low template samples – would further complicate profile interpretation. However, this study has shown that a consensus profile from a split single source sample contains considerably less information than a single profile from a non-split sample. It would, therefore, be preferable to build a statistical model that can be applied to the single LTDNA profile since this should provide the most information.

## Conclusions

Overall, this study has demonstrated that performing standard cycling STR typing on non-split DNA extracts will result in profiles with a higher percentage of total loci compared with the consensus profiling technique. Increasing the number of PCR cycles improves the sensitivity of the reaction compared with a standard cycle PCR. However, samples containing template amounts on the upper limits of what would be considered low template DNA may not actually benefit from the increased amplification because of the additional alleles, either drop in or stutter, that can appear in the profile. The repeat nature of the consensus profiling method does eliminate the problem of allele drop in seen with an increased number of PCR cycles, which has important implications for casework. However, consensus profiling also results in the least informative profiles due to increased allele or locus drop out. It also results in more ‘incorrect’ alleles in the individual profiles used to obtain the consensus profile as a result of increased stutter. Simply performing a single standard cycle PCR on the entire sample produced the most complete profiles when 100 pg of starting template are available for amplification. When only 25 pg of template are available, it would be beneficial to amplify the entire extract with an increased cycle PCR in terms of acquiring a profile with the most information possible. While this must be balanced against the possibility of drop in, it is important to realize that increased stutter alleles that are likely to appear in the split sample profiles are also incorrect alleles and are more likely to be reproducible than ‘random’ allele drop ins.

Performing a single STR reaction from the whole low template sample does eliminate any chance of repeating the profile. In this sense, consensus profiling may be preferred because the results are seen as repeatable. However, the impression of repeatability gained by the consensus profiling method must be balanced against the notable loss of information that occurs when a LTDNA sample is divided for amplification. While consensus profiling does have its benefits, the method may not be producing the most informative STR profiles for samples where the template amount is limited.

## Abbreviations

ADI, Allele drop in; ADO, Allele drop out; CE, Capillary electrophoresis; LCN, Low copy number; LDO, Locus drop out; LTDNA, Low template DNA; PCR, Polymerase chain reaction; PHR, Peak height ratio; RFU, Relative fluorescence unit; STR, Short tandem repeat.

## Competing interests

The authors declare that they have no competing interests.

## Authors’ contributions

KG carried out the molecular studies, data analysis and drafted the manuscript. AvD conceived of the study, participated in its design and coordination, and helped to draft the manuscript. Both authors read and approved the final manuscript.
